# Oscillatory correlates of auditory working memory examined with human electrocorticography

**DOI:** 10.1016/j.neuropsychologia.2020.107691

**Published:** 2021-01-08

**Authors:** Sukhbinder Kumar, Phillip E. Gander, Joel I. Berger, Alexander J. Billig, Kirill V. Nourski, Hiroyuki Oya, Hiroto Kawasaki, Matthew A. Howard, Timothy D. Griffiths

**Affiliations:** a Newcastle University Medical School, Newcastle Upon Tyne, Tyne and Wear NE2 4HH, UK; b Department of Neurosurgery, The University of Iowa, Iowa City, IA, 52242, USA; c UCL Ear Institute, University College London, London, WC1X 8EE, UK; d Iowa Neuroscience Institute, The University of Iowa, Iowa City, IA, 52242, USA; e Pappajohn Biomedical Institute, The University of Iowa, Iowa City, IA, 52242, USA; f Wellcome Centre for Human Neuroimaging, University College London, London, WC1N 3BG, UK

**Keywords:** Electrocorticography, Auditory working memory, Neurophysiology, Oscillatory activity, Hippocampus

## Abstract

This work examines how sounds are held in auditory working memory (AWM) in humans by examining oscillatory local field potentials (LFPs) in candidate brain regions. Previous fMRI studies by our group demonstrated blood oxygenation level-dependent (BOLD) response increases during maintenance in auditory cortex, inferior frontal cortex and the hippocampus using a paradigm with a delay period greater than 10s. The relationship between such BOLD changes and ensemble activity in different frequency bands is complex, and the long delay period raised the possibility that long-term memory mechanisms were engaged. Here we assessed LFPs in different frequency bands in six subjects with recordings from all candidate brain regions using a paradigm with a short delay period of 3 s. Sustained delay activity was demonstrated in all areas, with different patterns in the different areas. Enhancement in low frequency (delta) power and suppression across higher frequencies (beta/gamma) were demonstrated in primary auditory cortex in medial Heschl’s gyrus (HG) whilst non-primary cortex showed patterns of enhancement and suppression that altered at different levels of the auditory hierarchy from lateral HG to superior- and middle-temporal gyrus. Inferior frontal cortex showed increasing suppression with increasing frequency. The hippocampus and parahippocampal gyrus showed low frequency increases and high frequency decreases in oscillatory activity. This work demonstrates sustained activity patterns during AWM maintenance, with prominent low-frequency increases in medial temporal lobe regions.

## Highlights

• Local field potentials recorded in humans while they keep sound in working memory.
• Sustained increase in delta power observed in primary auditory cortex.
• Pattern of change in power in non-primary cortex depends on the hierarchical level.
• Hippocampus and parahippocampus showed increase in low frequency power.

## 1 Introduction

In this study we examine the process by which sounds are held in mind over seconds: auditory working memory (AWM). We use the term AWM to refer to the general process of keeping sound objects in mind as opposed to the specific process of phonological working memory to which it is sometimes applied. This general process is relevant to the analysis of auditory scenes in which auditory objects within the scene can remain constant, or come and go. Whilst a number of studies have demonstrated the involvement of early sensory areas in visual working memory (e.g. Pasternak and Greenlee, 2005; Postle, 2006), research examining the role of brain regions in auditory working memory is relatively limited. In particular, although research into neural mechanisms underlying verbal working memory is growing (e.g. Fiebach et al., 2006; 2006; Ivanova et al., 2018), the neural substrates of AWM in general are not well established.

Previous research into AWM used functional magnetic resonance imaging (fMRI), magnetoencephalography or positron emission tomography. With respect to sensory cortex, data from these studies are conflicting; whilst the majority of studies found increased activity in auditory cortex during the delay period (Gaab et al., 2003; Grimault et al., 2009; Kumar et al., 2016; Linke and Cusack, 2015), others showed suppression of activity (Linke et al., 2011; Zatorre et al., 1994). It should be noted that some of the original studies did not allow for separation of the delay period from encoding or retrieval periods of working memory, a factor which could explain discrepancies. With respect to a broader network involved in AWM maintenance, previous functional imaging has implicated the inferior frontal lobe and the hippocampus in maintenance of AWM (Kumar et al., 2016). However, that fMRI experiment used a long delay period to overcome the slow hemodynamic response (in order to demonstrate delay period activity ‘uncontaminated’ by encoding or retrieval activity) and may have engaged long-term memory mechanisms.

In this study we carried out LFP recordings from areas previously implicated in AWM maintenance using BOLD measurement. This allows examination of neural activity that is spatially and temporally precise in order to assess patterns of activity across frequency bands. The relationship between such LFP patterns and BOLD changes is complex. For example, studies of sensory cortex suggest a positive correlation between high-frequency LFP oscillations and BOLD (Mukamel et al., 2005; Oya et al., 2018) and negative correlation with lower (delta, theta) frequency bands, but in hippocampus this relationship does not seem to hold as a positive correlation between theta/delta frequency band and BOLD exists (Fig. 1 in Ekstrom, 2010). More fundamentally, the technique is a direct test of whether explicit sustained activity during maintenance occur in parts of the suggested brain network. This is not a given, based on considerable recent interest in ‘activity silent’ maintenance of working memory (Reynolds et al., 2009; for a recent review, see Sreenivasan & D'Esposito, 2019). Working with LFPs also allows us to use a paradigm with a short delay period during which activity can only be interpreted on the basis of working memory.

**Fig. 1:**
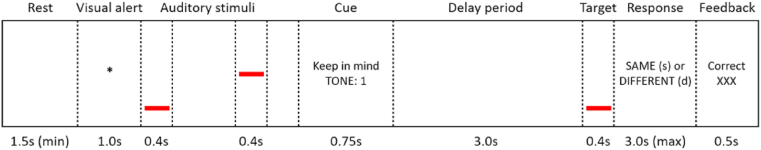
Paradigm for working memory task. Subjects were cued to maintain a particular tone for a period of 3 s following the presentation of two different tones. They were then asked to compare the maintained tone with a target tone, and given feedback as to whether they were correct, followed by a rest period.

## 2 Methods

### 2.1 Subjects

Six neurosurgical patients were studied (all six male, right-handed with left language dominance, age 17–39 years old); five were within the low to high average intellect range with normal working memory, while one patient (L275) was cognitively impaired and deficient in working memory on neuropsychological assessment. They had medically refractory epilepsy for which electrocorticography was required to identify seizure foci prior to potential resection surgery. Electrode contacts in the region of the seizure focus were not included in further analysis. Research protocols were examined and approved by the University of Iowa Institutional Review Board.

### 2.2 Electrodes and recording setup

Recordings were made from subjects in an electromagnetically shielded facility. Intracranial data were recorded from the following electrode arrays implanted on the right ride (N = 3, as indicated by the letter prefix ‘R’ in the subject code) and the left (N = 3, prefix code ‘L’): an 8-contact depth electrode (5 mm center-to-center distance) placed along the axis of Heschl’s gyrus (Howard et al., 1996; Nagahama et al., 2018; Reddy et al., 2010); a 96-contact grid (5 mm center-to-center distance) covering a large area of the temporal lobe (and in one subject portions of the inferior frontal gyrus; IFG); multi-contact grids (16 or 32 contacts) overlying inferior frontal cortex; and a strip electrode (12 contacts) placed medially along the anterior ventral portion of the temporal lobe. All electrodes (Ad-Tech Medical Instrument, Racine, WI and PMT Corporation, Chanhassen, MN) were digitized using a Tucker Davis Technologies (Alachua, FL) RZ2 system and referenced to a subgaleal electrode. Data were acquired using a 0.7–800 Hz bandpass filter (sampled at 2034 Hz) at 16-bit resolution. Electrode placement was selected based purely on clinical considerations for seizure monitoring. Regions of interest (ROIs) for the purposes of analysis were guided by our previous construction of an auditory working memory network (Kumar et al., 2016), but with greater spatial precision than was available when using fMRI. These included posteromedial Heschl’s gyrus (HGpm), anterolateral Heschl’s gyrus (HGal), superior temporal gyrus (STG), middle temporal gyrus (MTG), inferior frontal gyrus (IFG), hippocampus (HC) and parahippocampal gyrus (PHG).

### 2.3 Stimuli and procedure

Auditory stimuli were delivered diotically using Etymotic ER4B earphones with custom-made earmolds. Sounds were set at a level that was confirmed to be comfortable for each subject. The experimental procedure was based on that used in Kumar et al. (2016) shown in Fig. 1. For each trial, subjects were presented with a visual alert which indicated the start of a trial. Following a 1 s delay, two tones (400 ms duration, 500 ms for subject L275, gated with a cosine window of 10 ms at the onset and offset), selected randomly, one from each of the two sets consisting of 8 tones, logarithmically sampled from a low frequency (between 300 and 570 Hz) or high frequency (between 2000 and 2800 Hz) range. A silent inter-stimulus interval of 750 ms (1 s for L275) separated the two tones. After another delay of 250 ms (500 ms for L275), a visual cue was presented for 750 ms (1.5 s for L275), instructing the subject to remember either the first or the second tone. This was followed by a 3000 ms period during which the subjects were required to remember the tone, referred to as the “delay period”. Finally, subjects were presented with a target tone which on half of the trials was the same tone as they were asked to remember, and on the other half of trials was either + or – 20% of the cued tone frequency. They were then visually instructed to respond on the keyboard whether this target tone was the same or different to the tone they had been asked to remember. They were given feedback as to whether they were correct or not, which was followed by a short rest period (4500–5500 ms) prior to the start of the subsequent trial. The total trial duration was 14.25–16.7 s. Subjects completed up to 160 trials in total.

### 2.4 Imaging

A high-resolution T1-weighted structural MRI of the brain was acquired for each subject before and after electrode implantation. Images were acquired from a 3T Siemens TIM Trio scanner with a 12-channel head coil (MPRAGE: 0.78 × 0.78 mm, slice thickness 1.0 mm, TR = 2.530 s, TE = 3.520 ms, average of two). To determine the location of recording contacts on the preoperative structural MRI, these images were coregistered to post-implantation structural MRIs using a 3D linear registration algorithm (Functional MRI of the Brain Linear Image Registration Tool; Jenkinson et al., 2012) and custom Matlab v.9.0 (MathWorks, Natick, MA) scripts using guidance from computed tomography (CT) scans (in-plane resolution 0.51 × 0.51 mm, slice thickness 1.0 mm). When possible, results were also compared with intraoperative photographs to ensure reconstruction accuracy. To be included, all contacts were confirmed to be in grey matter within each ROI. Each individual’s brain was coregistered to the ICBM152 template (Fonov et al., 2011) to estimate contact location in MNI template space.

### 2.5 Data processing and analysis

Data were analysed offline using custom-written Matlab scripts and the EEGLab toolbox (Delorme and Makeig, 2004). In order to probe successful working memory processes, only trials where the subject responded correctly were included. Data were downsampled to 500 Hz and trials were epoched, with times ranging from 2000 ms prior to the initial visual alert to 11,000 ms after. Line noise was removed by implementing the demodulated band transform (Kovach and Gander, 2016). Data were further denoised by removing the first principal component from the singular value decomposition of the highpass-filtered data (>160 Hz).

Morlet-based wavelet analyses were performed to examine changes in frequency-specific activity over time. Trials were first rejected using automated artifact rejection, based on kurtosis and large common amplitudes across electrodes (Delorme et al., 2007). Remaining data were processed in sliding windows, using a minimum of 3 cycles per window for the lowest frequency, increasing linearly with increasing frequency. Wavelet analyses were calculated between 2 and 150 Hz. Time-frequency data were normalized to the average power across trials. Statistical significance at every time-frequency point was tested using bootstrapping. The surrogate data for bootstrap was obtained by shuffling the power values 200 times during the baseline time period (−1000 ms to −100 ms). A significance threshold of 0.01 (two-tailed) was set for the purposes of displaying the data, relative to the bootstrapped values. Band power during the delay period was calculated by taking the mean power across time and frequency within canonical frequency bands, omitting the first and last 100 ms to account for the width of the Gaussian window. These were delta (2–4Hz), theta (4–8 Hz), alpha (8–13 Hz), beta (13–30 Hz), low gamma (30–70 Hz) and high gamma (70–150 Hz). To reduce the dimensionality of the data, time-frequency data were selected from the electrode exhibiting the strongest absolute response (i.e. regardless of directionality) within any frequency band, which was confirmed to be representative of surrounding electrodes, for each ROI.

To further examine the dynamics of the oscillatory activity, cluster-based permutation tests (Maris and Oostenveld, 2007) were run, comparing the power in each canonical frequency range during the delay period to the average power during the baseline period, for each ROI. This involved running two-tailed, paired *t*-tests at every timepoint. Data were clustered on the basis of temporal adjacency, with a pre-designated threshold of *p* < 0.05 set for determining the *t*-values required. The largest summed *t*-value cluster was used as the test statistic. Data were then shuffled between baseline and the delay period and this method was repeated for all possible permutations, to create a distribution of the largest summed *t*-value clusters. Summed *t*-value clusters from the actual data that fell outside the 95% confidence interval of this distribution were considered significant.

## 3 Results

**Fig. 2:**
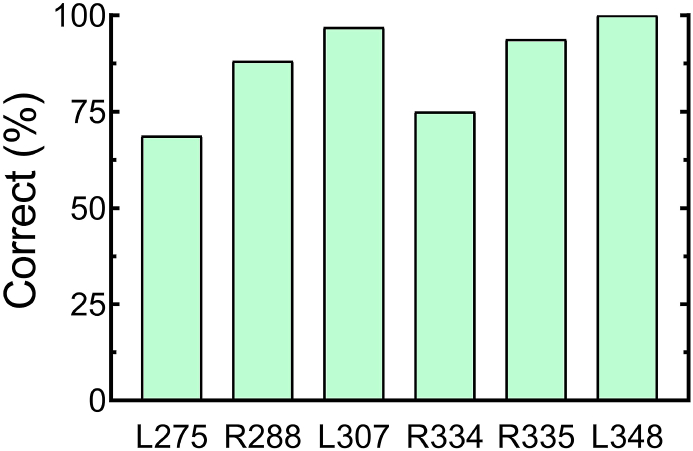
Behavioral performance of all subjects in the task.

Percentage accuracy of the subjects in the task varied from 67% to 99% (Fig. 2). All subjects performed above chance level (chi-square test, p < 0.05).

**Fig. 3:**
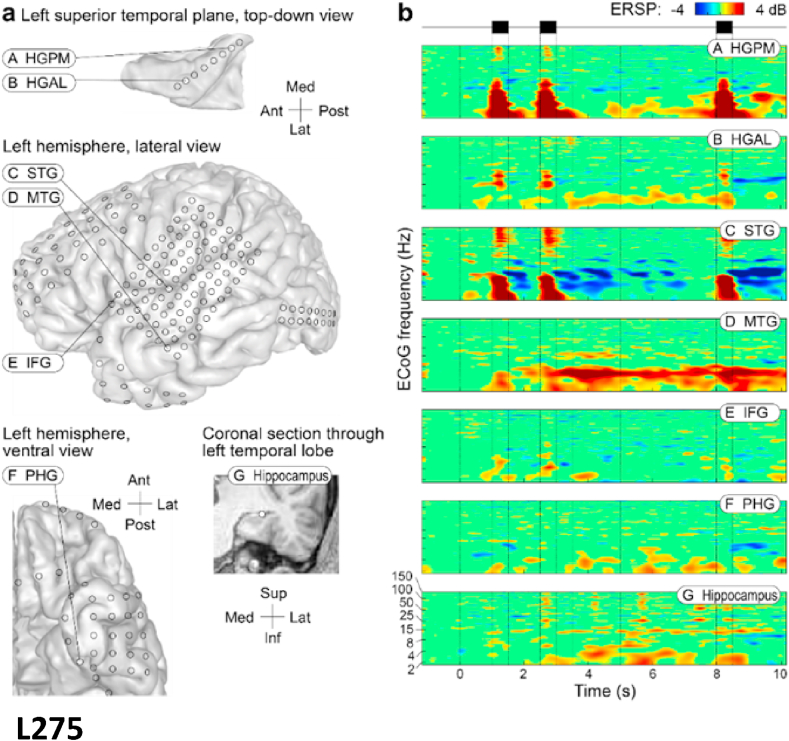
Example intracranial data from a single patient (L275). **a.** Location of implanted electrodes, with an 8-channel depth electrode along the axis of HG, a single 96-channel grid over STG, MTG and IFG, a strip electrode overlying the PHG and a depth electrode into HC. **b.** Corresponding event-related spectral perturbations (ERSPs) recorded from the ROIs during the working memory task.

**Fig. 4:**
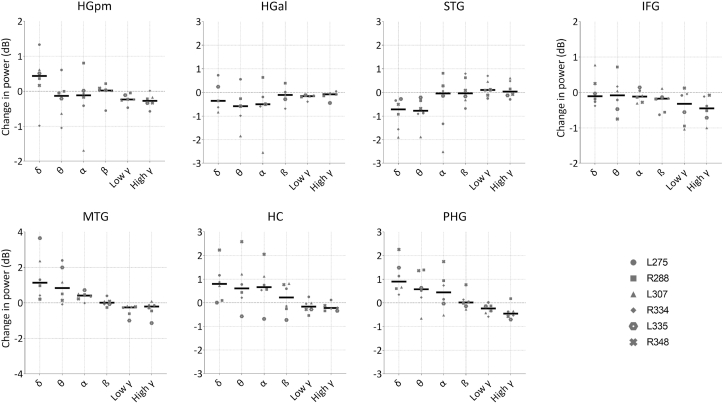
Change in power during the delay period, for each canonical frequency band and each ROI, plotted across subjects (*n* = 6). Thick coloured lines represent median across subjects. Grey markers indicate individual subject data. Axes are scaled to the maximum value for each ROI.

Example event-related spectral perturbations (ERSPs) from one subject are shown in Fig. 3. The ERSPs for remaining subjects are included as supplementary figures (Supplementary Figures 1-5). Activity during the delay period is described below for each ROI. Data across subjects are summarized as median changes in power (dB) relative to baseline. These data are shown in Fig. 4.

### 3.1 Posteromedial Heschl’s gyrus (HGpm)

During the delay period, increases in delta band power were evident in HGpm in five of the six subjects, with a median value of 0.44 dB. Activity in other frequency bands was either unchanged across subjects (beta = 0.02 dB) or decreased to varying degrees (theta = −0.13; alpha = −0.12 dB; low gamma −0.24 dB; high gamma −0.27 dB).

**Fig. 5:**
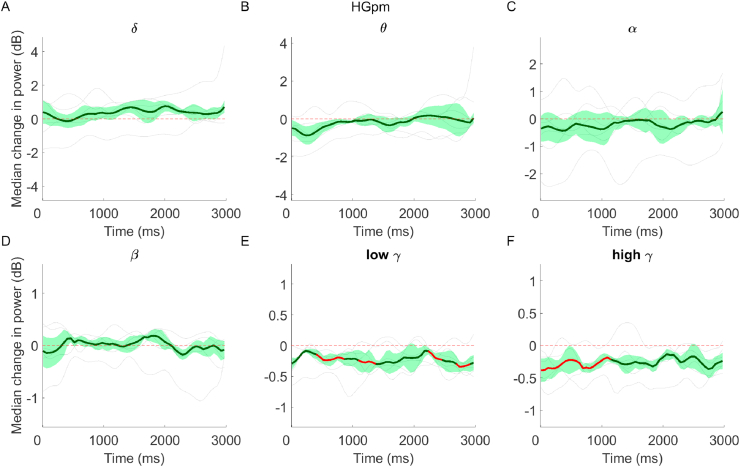
Median change in power across subjects, relative to baseline, plotted across the entire delay period for HGpm. Time referenced to start of delay period. Green shading indicates median absolute deviation (MAD), light grey lines show individual subject data, whilst red areas highlight periods of significance, determined using cluster-based permutation tests with an alpha level of 0.05. (For interpretation of the references to colour in this figure legend, the reader is referred to the Web version of this article.)

Data were also plotted over the whole delay period, to examine the temporal dynamics of oscillatory activity (Fig. 5). With the exception of the one subject who exhibited a decrease in activity, the increases in delta band activity were not restricted to a particular period but were distributed across the whole epoch after the first 500 ms. In contast, a transient decrease in theta band activity occurred that was restricted to the first ~750 ms of the delay period in all but one subject (Fig. 5B), whilst decreases in low and high gamma and, to a lesser extent, alpha band, were sustained throughout the period (Fig. 5C, E, and 5F). However, when examined on a moment-by-moment basis, only the decreases in low and high gamma during the delay period were significantly different from baseline (cluster-based permutation test; *p* < 0.05). Note that the sharp increase of power in delta/theta bands towards the end of the delay period is due to the long analysis window for these bands, which overlaps with the upcoming sound period.

### 3.2 Anterolateral Heschl’s gyrus (HGal)

Median activity in HGal was decreased in all frequency bands: delta (−0.36 dB), theta (−0.58 dB), alpha (−0.50 dB), beta (−0.11 dB), low gamma (−0.16 dB) and high gamma (−0.08 dB). Analysis of activity over time revealed that decreases in delta through alpha band activity were generally restricted to the first ~750 ms of the delay period (in all but one subject). As with HGpm, decreases in low gamma and high gamma were evident throughout the whole period, although these were only significant across subjects for low gamma band (Supplementary Figure 6).

### 3.3 Superior temporal gyrus (STG)

Large decreases in delta (−0.71 dB) and theta activity (−0.78 dB) were evident in STG during the delay period. Median alpha, beta and high gamma band activity was relatively unchanged (−0.04 dB,-0.04 dB and 0.03 dB respectively), although this was variable across subjects. Slight increases occurred in low gamma (0.1 dB). When examined over time across subjects, significant decreases in delta and theta were evident during the first 1000 ms of the delay period (cluster-based permutation test, *p* < 0.05; Supplementary Figure 7).

### 3.4 Medial temporal gyrus (MTG)

Clear increases in delta (1.10 dB) and theta activity (0.83 dB) were present in MTG during the delay period. When examined across subjects and time, these changes were sustained throughout the whole period but only reached significance sporadically (Supplementary Figure 8). More moderate increases were also evident in alpha (0.41 dB), whilst low and high gamma were decreased (−0.26 dB and −0.21 dB, respectively), and beta band activity was unchanged (0dB). Decreases in low gamma were significant for the first ~600 ms but power in this band remained suppressed for the duration of the delay period.

### 3.5 Inferior frontal gyrus (IFG)

**Fig. 6:**
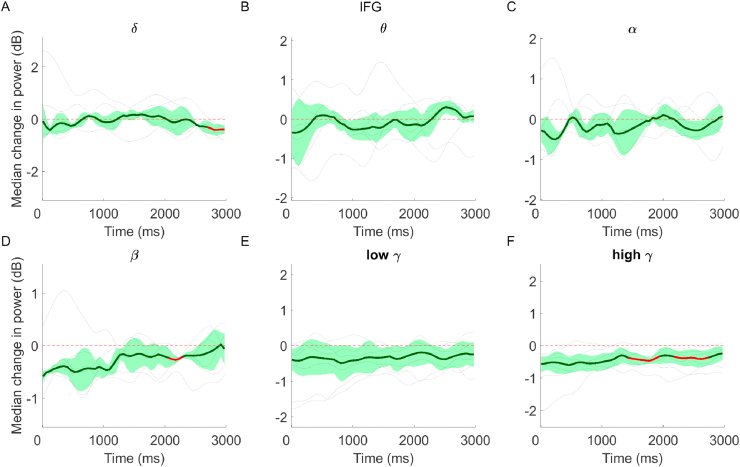
Median change (±MAD) in power relative to baseline, across subjects and over time, in IFG. Red areas highlight periods of significance. Single subject data are overlaid (light grey lines). (For interpretation of the references to colour in this figure legend, the reader is referred to the Web version of this article.)

Median band power in IFG decreased with increasing frequency. Slight decreases were evident in delta (−0.11 dB), theta (−0.08 dB) and alpha (−0.12 dB) bands, and larger decreases were present in beta (−0.17 dB), low gamma (−0.32 dB) and high gamma bands (−0.45 dB). Although delta band activity was unchanged when averaging band power across the whole delay period, there was a significant decrease evident in the final ~300 ms (Fig. 6A). Decreases in beta were sustained for the majority of the delay period, although these showed an upward trend towards the final 750 ms (Fig. 5D). Gamma band activity was suppressed throughout the epoch, but only reached significance across subjects for high gamma in the second half of the delay period (Fig. 6E–F).

### 3.6 Hippocampus (HC)

**Fig. 7:**
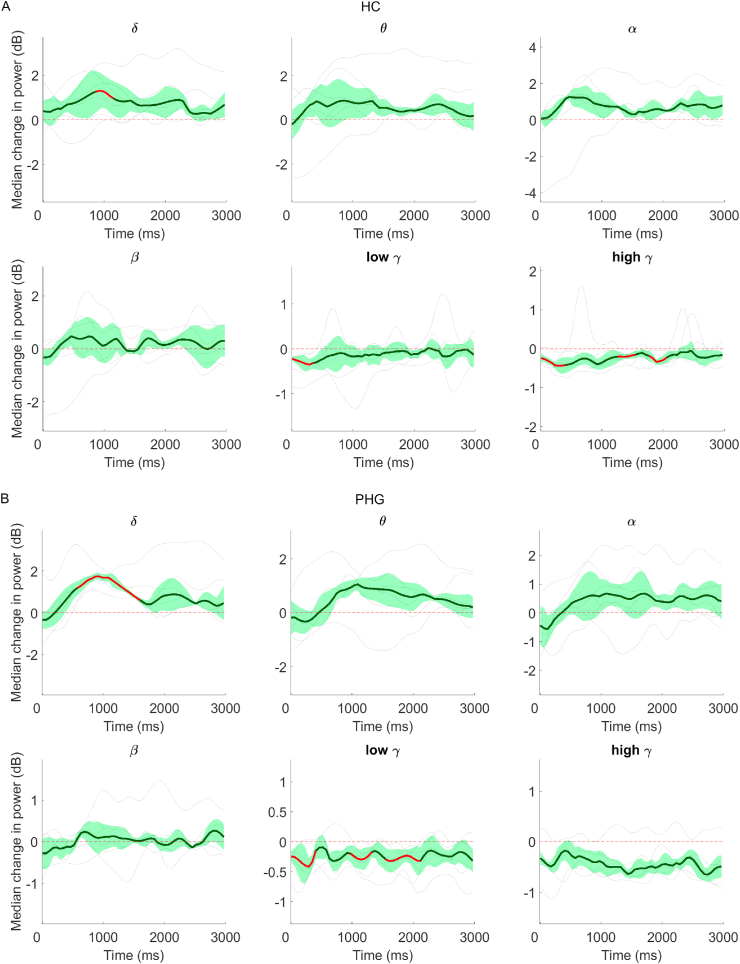
Median change (±MAD) in power across subjects, relative to baseline, plotted across the entire delay period for HC **(A)** and PHG **(B)**. Red areas highlight periods of significance. Single subject data are overlaid (light grey lines). (For interpretation of the references to colour in this figure legend, the reader is referred to the Web version of this article.)

Across subjects, clear increases in hippocampus oscillatory activity were evident in delta (0.80 dB), theta (0.61 dB), alpha (0.66 dB) and - to a lesser extent - beta band (0.22 dB). Median activity in low and high gamma bands decreased (−0.17 dB and −0.22 dB, respectively). Temporal presentation of the data revealed that the increases in delta band activity were sustained throughout the delay period whilst other bands generally showed a build-up within the first 1000 ms and then remained sustained (Fig. 7A). Due to variability across subjects, these increases did not generally reach significance, other than for a short section of the delay period for delta activity. Low gamma activity was significantly reduced across subjects during the first ~500 ms of the delay period, whilst significant decreases in high gamma activity were periodic and not sustained for the whole period.

### 3.7 Parahippocampal gyrus (PHG)

Clear increases were evident in delta band activity in all subjects (0.90 dB). Similar to HC, increases were also evident in theta (0.57 dB) and alpha bands (0.44 dB), whilst decreases were present in low gamma (−0.24 dB) and high gamma (−0.46 dB). Median beta band activity was relatively unchanged (0.01 dB). Increases in low frequency activity showed a build-up within the first ~750 ms and were then evident for the remainder of the delay period when examined over time (Fig. 7B). Due to some variability across subjects, these increases were significant only from 500 to 1500 ms for delta band activity. Decreases in both low and high gamma band activity were generally sustained for the entire delay period.

## 4 Discussion

We have demonstrated activity during auditory working memory maintenance in all three candidate areas: auditory cortex, inferior frontal cortex and medial temporal lobe. Delta power increases and suppression across higher frequencies were found in primary auditory cortex in medial Heschl’s gyrus (HG) whilst non-primary cortex showed patterns of activation and suppression that altered at different levels of the auditory hierarchy from lateral HG to superior and middle temporal gyrus. Inferior frontal cortex showed increasing suppression with increasing frequency. The hippocampus and parahippocampal gyrus showed low-frequency increases and high-frequency decreases in oscillatory activity.

### 4.1 Delay activity in primary auditory cortex

Above-baseline delta power was previously identified in auditory cortex while subjects kept tone frequencies in mind for a brief interval (0.5–3s; Herbst and Obleser, 2019). While that scalp EEG study could not distinguish between auditory fields, the delta power increase we report is specific to primary cortex in posteromedial Heschl’s gyrus. Decreases in power for other low frequencies (theta and alpha) and high frequencies (gamma and high gamma) were observed in the primary auditory cortex. In our previous study, we demonstrated an increase in BOLD response in the same region during a longer delay period (Kumar et al., 2016). This can be reconciled with our current findings by recalling that increase in BOLD signal can be either due to decrease in low frequencies (delta, theta, alpha) and/or increase in gamma/high gamma power. Also, in terms of predicting a BOLD signal, the different frequency bands are not weighted equally. For example, theta and alpha frequencies have a higher (negative) weightage than delta band and (positive) weightage of gamma/high gamma is lower than (negative) weightage of theta/alpha bands (Mukamel et al., 2005; Zumer et al., 2010). There is also some uncertainty regarding the lowest frequency up to which BOLD has a negative correlation. While Mukamel et al. (2005) show this to be beta band, other studies e.g. Zumer et al. (2010) show negative correlation of BOLD extends up to lower gamma band (~50Hz). Therefore, decreases in theta/alpha (and possibly gamma) band combined with their stronger weightings could explain the increase in BOLD response observed in our previous work (Kumar et al., 2016).

Recent theoretical and empirical work points to short-term updating of synaptic weights in sensory cortex as one mechanism for working memory maintenance (Masse et al., 2019; Miller et al., 2018; Sreenivasan & D'Esposito, 2019; Wolff et al., 2017; Wolff et al., 2020). Such effects are described as activity-silent: they are not directly measurable as changes in neural firing or local field potentials but may be inferred through their impact on neural responses to brief task-irrelevant impulse stimuli presented during the maintenance period (Wolff et al., 2017; Wolff et al., 2020). While this approach holds promise, our data show that keeping the frequency of a tone in mind can in fact generate sustained oscillatory signatures in auditory cortex and beyond. Further work is required to establish the relationship between the LFP delta increases that are seen here, neuronal activity, and measures of the synaptic ‘trace’ that might maintain AWM.

### 4.2 Delay activity in higher-order lateral temporal cortex

Non-primary auditory cortex in HGal and STG did not show the same delta power increase as HGpm; indeed power in these regions was more suppressed across low-frequencies (delta-alpha). This distinction between oscillatory signatures in primary and non-primary auditory fields is similar to that observed during active listening to speech (Billig et al., 2019). In our previous fMRI study (Kumar et al., 2016), we showed a greater increase in BOLD signal (compared to primary auditory cortex) as we moved up the hierarchy to lateral HG and planum temporale. This is consistent (as per the discussion above) with a stronger decrease in power of all low frequencies (delta, theta, alpha) in HGal and with decreased power of delta/theta band in STG. There is not much change in the power of gamma/high gamma band in these regions.

Moving up the hierarchy, a contrasting and robust above-baseline increase in delta and theta power was observed in MTG. Interestingly, this oscillatory pattern is more similar to that in the medial temporal lobe (PHG/HC) than in hierarchically earlier auditory sites. We had no prior expectations of activity in this region. MTG is known to represent high-level sound categories during imagery (Linke and Cusack, 2015) and to be involved in semantic processing more generally (Davey et al., 2016; Hickok and Poeppel, 2004). Despite the simple nature of the pure tone stimuli, it is possible that subjects tried to apply semantic labels in performing the task.

### 4.3 Delay activity in inferior frontal gyrus

Above-baseline BOLD activity and PET regional cerebral blood flow have been reported in inferior frontal gyrus during the maintenance and comparison of pure tones (Zatorre et al., 1994; Kumar et al., 2016) and amplitude modulation rate (Uluc et al., 2018). The present study demonstrates suppression of LFP activity in inferior frontal cortex that is greatest at high frequency and a less pronounced decrease in power of lower frequencies. If we use the same rationale as used for auditory cortex in the previous sections, then the oscillatory pattern of IFG would predict a decrease rather than an increase in BOLD signal, which is inconsistent with our previous fMRI work. However, two points need to be kept in mind before this conclusion is drawn. First, the relation between oscillatory power and BOLD in the IFG may not be same as in sensory cortex. For example, while beta band power (20–30Hz) is positively correlated with BOLD in the auditory cortex (Mukamel et al., 2005), power in the same frequency band is highly negatively correlated with BOLD signal in frontal regions (Hanslmayr et al., 2011). Second, IFG coverage in the current study does not the overlap with the region identified in the fMRI study, which is much more ventral (frontal operculum bordering on anterior insula).

### 4.4 Delay activity in the medial temporal lobe

Although our previous fMRI work identified hippocampal activity in support of auditory working memory (Kumar et al., 2016), the prolonged delay period in that study may have engaged a long-term or episodic hippocampal store (Scoville and Milner, 1957; Squire, 1992). In the current experiment, the temporal resolution of electrophysiological recordings allowed us to reduce the interval to 3 s and still dissociate encoding from delay activity. The pronounced power increases observed in (para)hippocampal delta-theta here are consistent with the fMRI findings (Kumar et al., 2016), given the positive correlation between low frequency power and the BOLD signal in hippocampus (Ekstrom, 2010). They are also reminiscent of hippocampal signatures of successful spatial and episodic encoding of memories tested over longer timeframes (Cornwell et al., 2008; Lega et al., 2012). Theta oscillations and the coupling of their phase with firing and high frequency activity have also been associated with visual and phonological working memory beyond the medial temporal lobe (Raghavachari et al., 2001; Canolty et al., 2006).

While some previous work emphasises a role for the medial temporal lobe in working memory when maintaining complex, conjunctive, or novel features (reviewed in Sreenivasan & D'Esposito, 2019), the tone frequencies we manipulated form a fundamental sound dimension. Rodent work has shown tuning for this feature in hippocampus and entorhinal cortex when it is task relevant (Aronov et al., 2017) although we do not know whether this is the case in humans. On the other hand, cells in hippocampus that fire in a fixed sequence to span a delay period have been identified both in rodents (Pastalkova et al., 2008; MacDonald et al., 2013) and humans (Umbach et al., 2020). Their ability to support working memory may depend on input from adjoining parahippocampal fields (Suh et al., 2011; Robinson et al., 2017).

## 5 Conclusions and outlook

This work demonstrates patterns of activity during AWM that are sustained during a delay interval, with prominent low-frequency increases in medial temporal lobe regions. Establishing the relationship between this medial temporal activity and that in areas more routinely associated with AWM, including inferior frontal gyrus and auditory cortex, will be an important next step. The hippocampus and inferior frontal gyrus may help keep sensory traces active in auditory cortex via ongoing top-down signaling, or alternatively maintain independent representations at different degrees of abstraction from the stimulus (Kumar et al., 2016; Huang et al., 2016). Future studies that draw on effective connectivity and pattern similarity analyses will be key to distinguishing between these and other accounts.

## Author credits

Sukhbinder Kumar: Conceptualization, Methodology, Software, Formal analysis, Writing – review & editing. Phillip E. Gander: Conceptualization, Methodology, Formal analysis, Investigation, Visualization, Writing – review & editing. Joel I. Berger: Methodology, Software, Formal analysis, Visualization, Investigation, Writing – original draft, Writing – review & editing. Alexander J. Billig: Methodology, Formal analysis, Writing – review & editing. Kirill V. Nourski: Investigation, Writing – review & editing. Hiroyuki Oya: Investigation, Writing – review & editing. Hiroto Kawasaki: Investigation, Writing – review & editing. Matthew A. Howard III: Resources, Writing – review & editing, Supervision, Resources, Funding acquisition Timothy D. Griffiths: Conceptualization, Writing – review & editing, Supervision, Project administration, Funding acquisition

## Declaration of competing interests

None
