# Supplementary material for: Oscillatory correlates of auditory working memory examined with human electrocorticography
Source: Neuropsychologia. 2021 Jan 8;150:107691. doi: 10.1016/j.neuropsychologia.2020.107691 (PMC7884909; doi:10.1016/j.neuropsychologia.2020.107691)
Supplement: Multimedia component 1 [file mmc1.docx]

**Supplementary Figures**

**
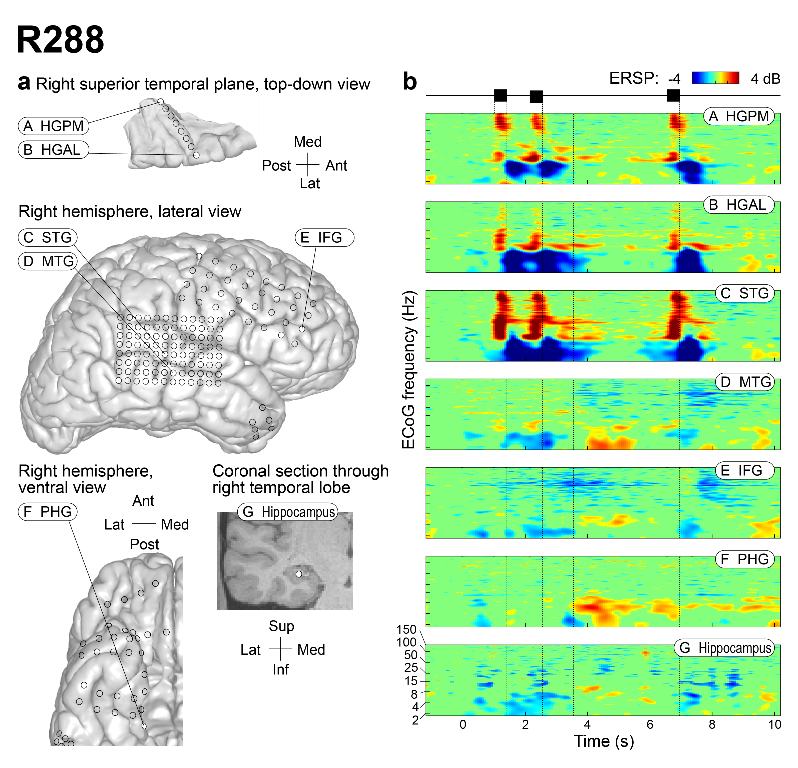
**

**Figure S1.** ERSP data for subject R288. **A.** Location of implanted electrodes. **B.** Corresponding event-related spectral perturbations (ERSPs) recorded from the ROIs during the working memory task.

**
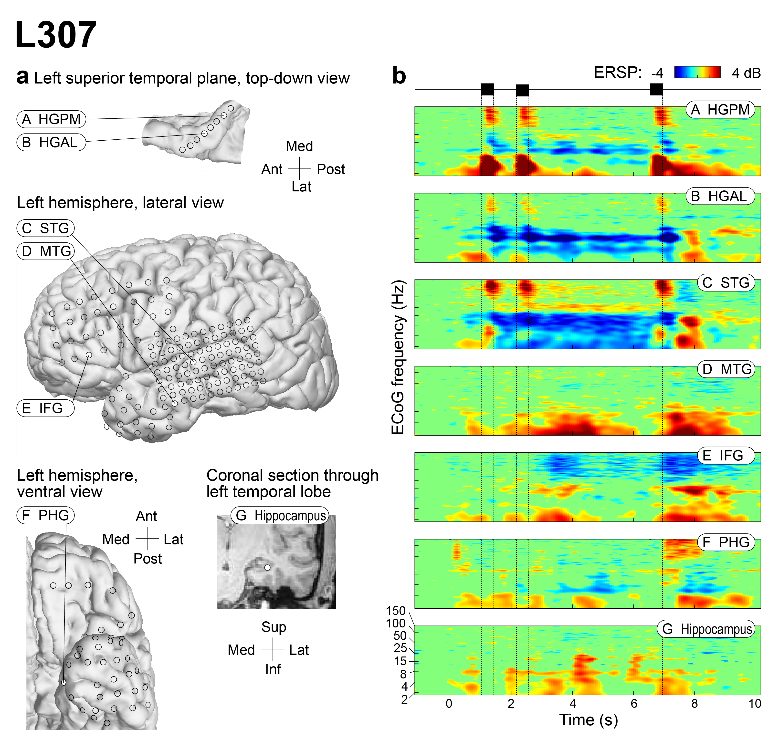
**

**Figure S2.** ERSP data for subject L307. **A.** Location of implanted electrodes. **B.** Corresponding event-related spectral perturbations (ERSPs) recorded from the ROIs during the working memory task.

**
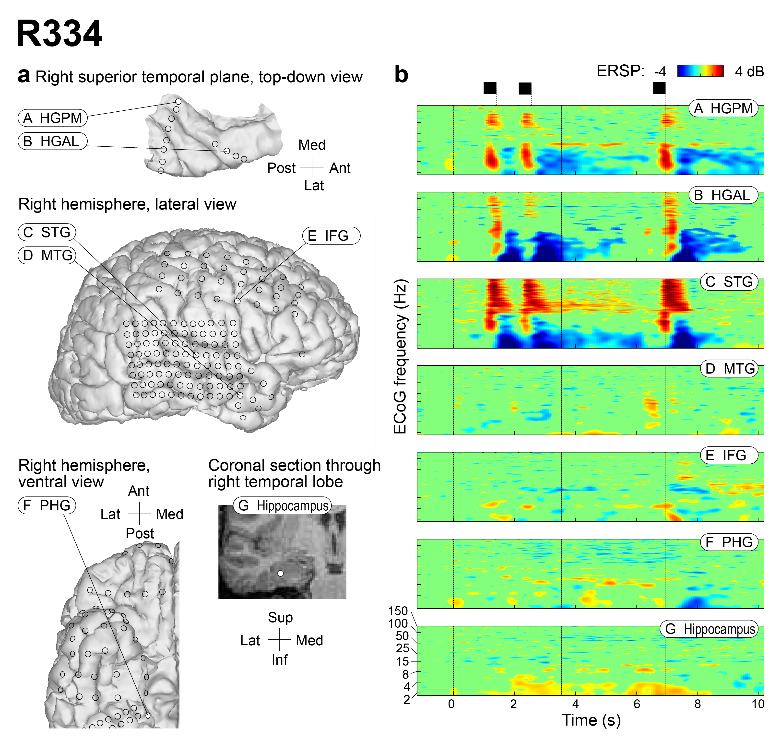
**

**Figure S3.** ERSP data for subject R334. **A.** Location of implanted electrodes. **B.** Corresponding event-related spectral perturbations (ERSPs) recorded from the ROIs during the working memory task.

**
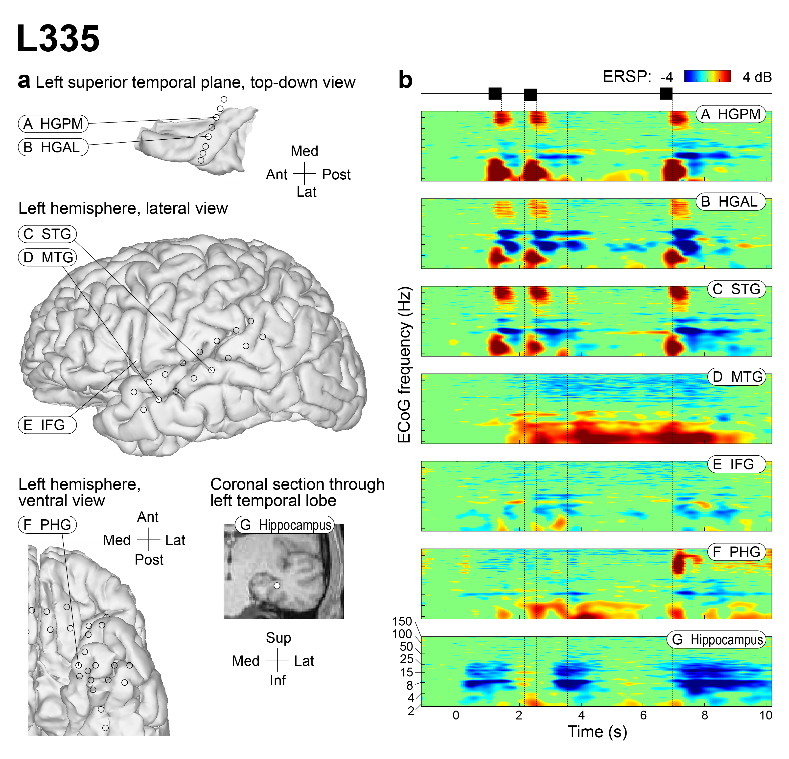
**

**Figure S4.** ERSP data for subject L335. **A.** Location of implanted electrodes. **B.** Corresponding event-related spectral perturbations (ERSPs) recorded from the ROIs during the working memory task.

**
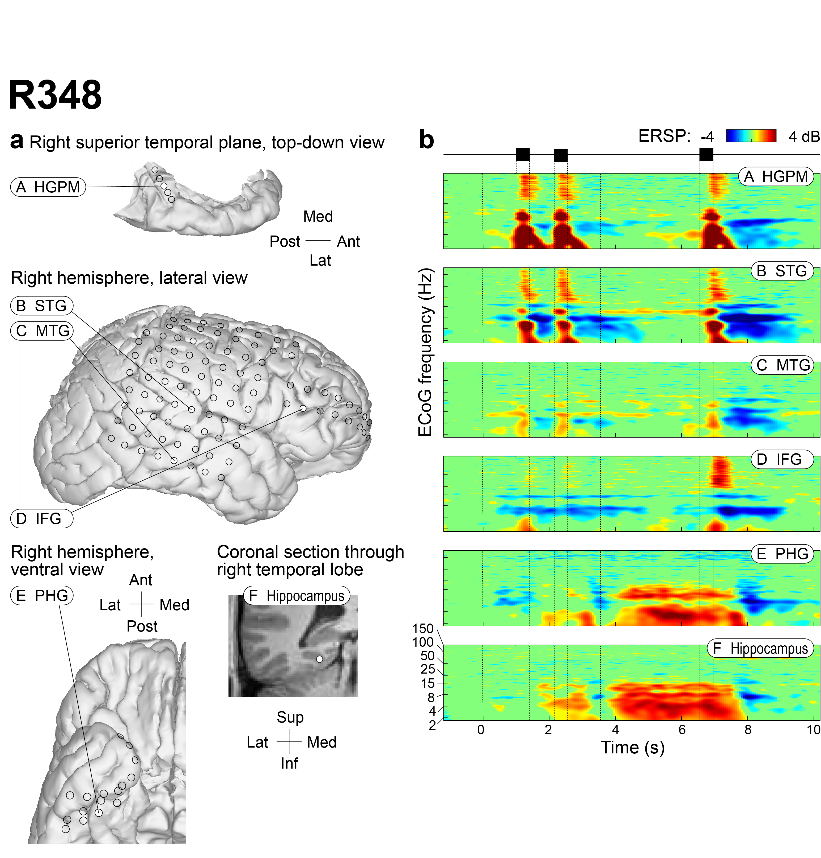
**

**Figure S5.** ERSP data for subject R348. **A.** Location of implanted electrodes. **B.** Corresponding event-related spectral perturbations (ERSPs) recorded from the ROIs during the working memory task.

**
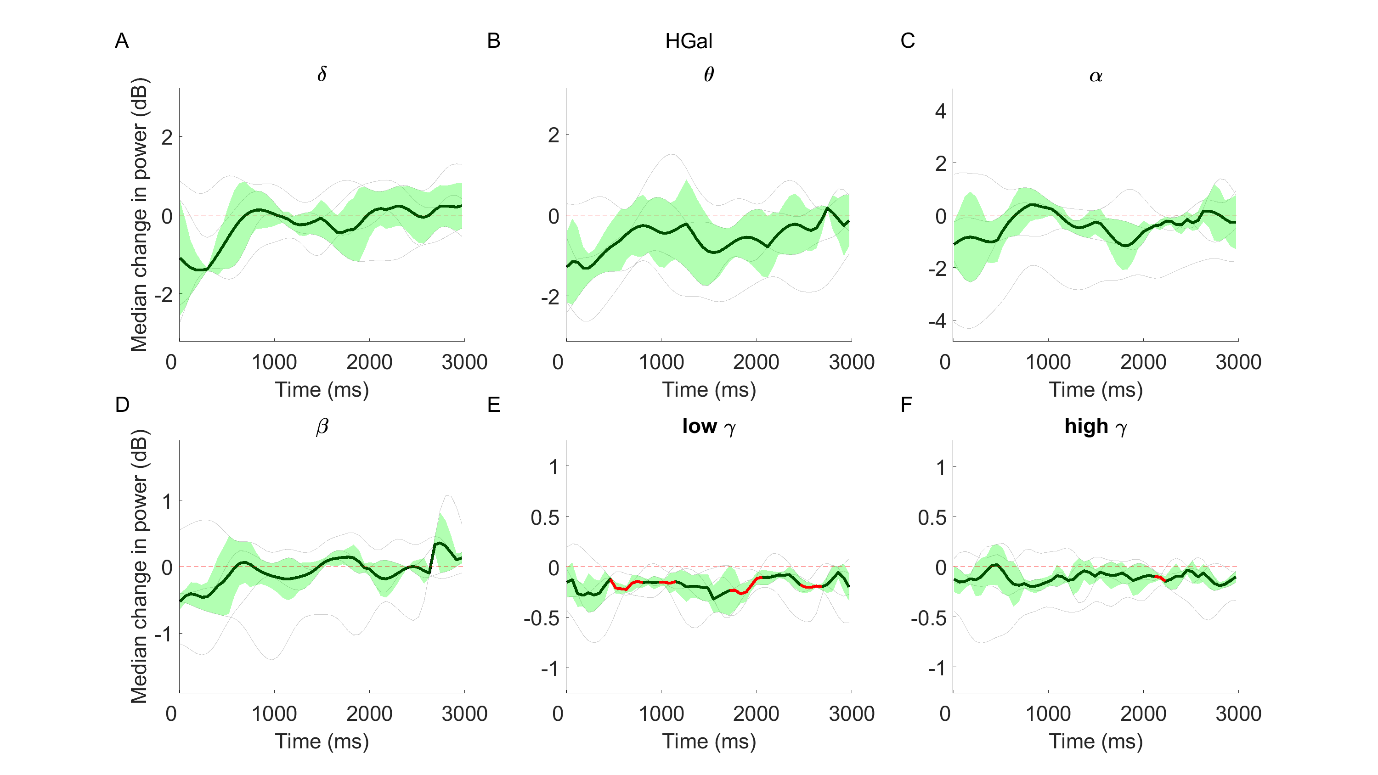
**

**Figure S6.** Median change (± MAD) in power across subjects, relative to baseline, plotted across the entire delay period for HGal. Red areas highlight periods of significance.

**
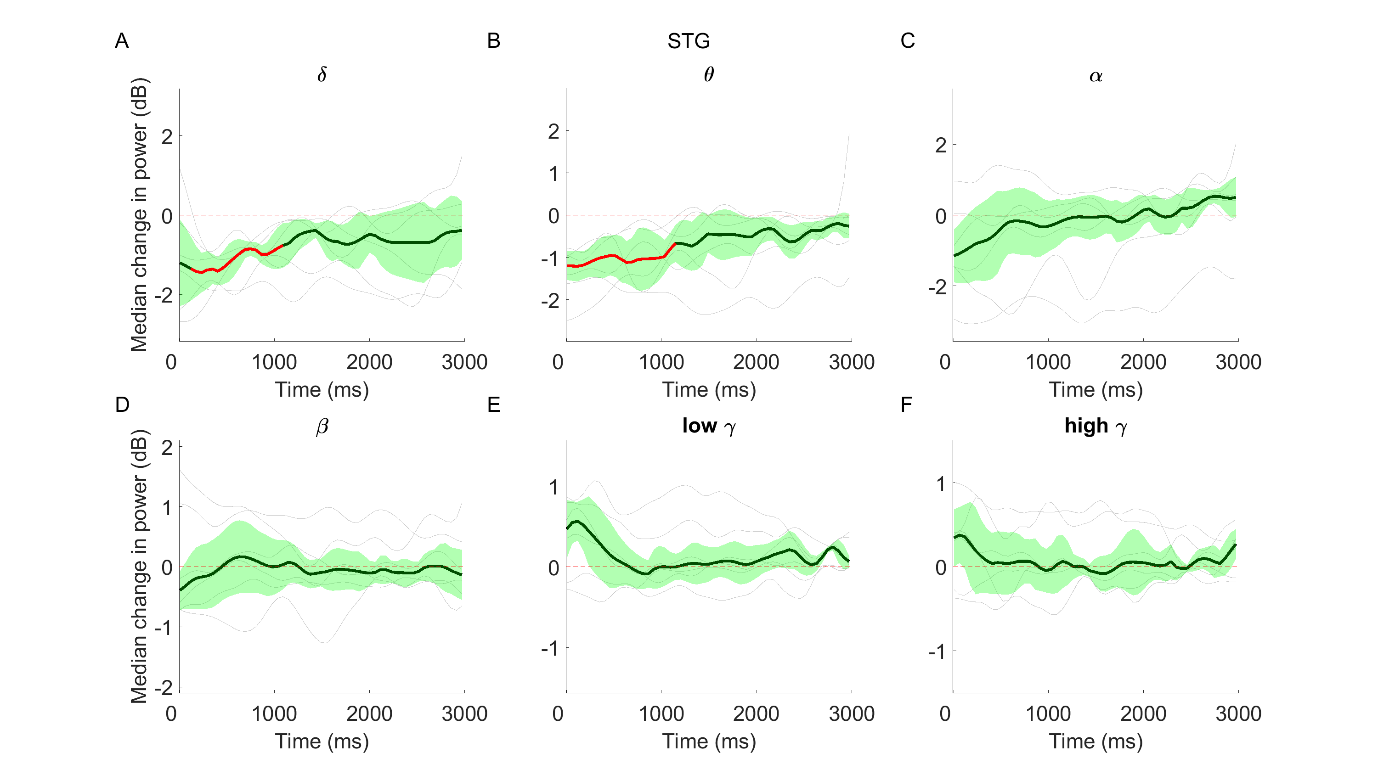
**

**Figure S7.** Median change (± MAD) in power across subjects, relative to baseline, plotted across the entire delay period for STG. Red areas highlight periods of significance.

**
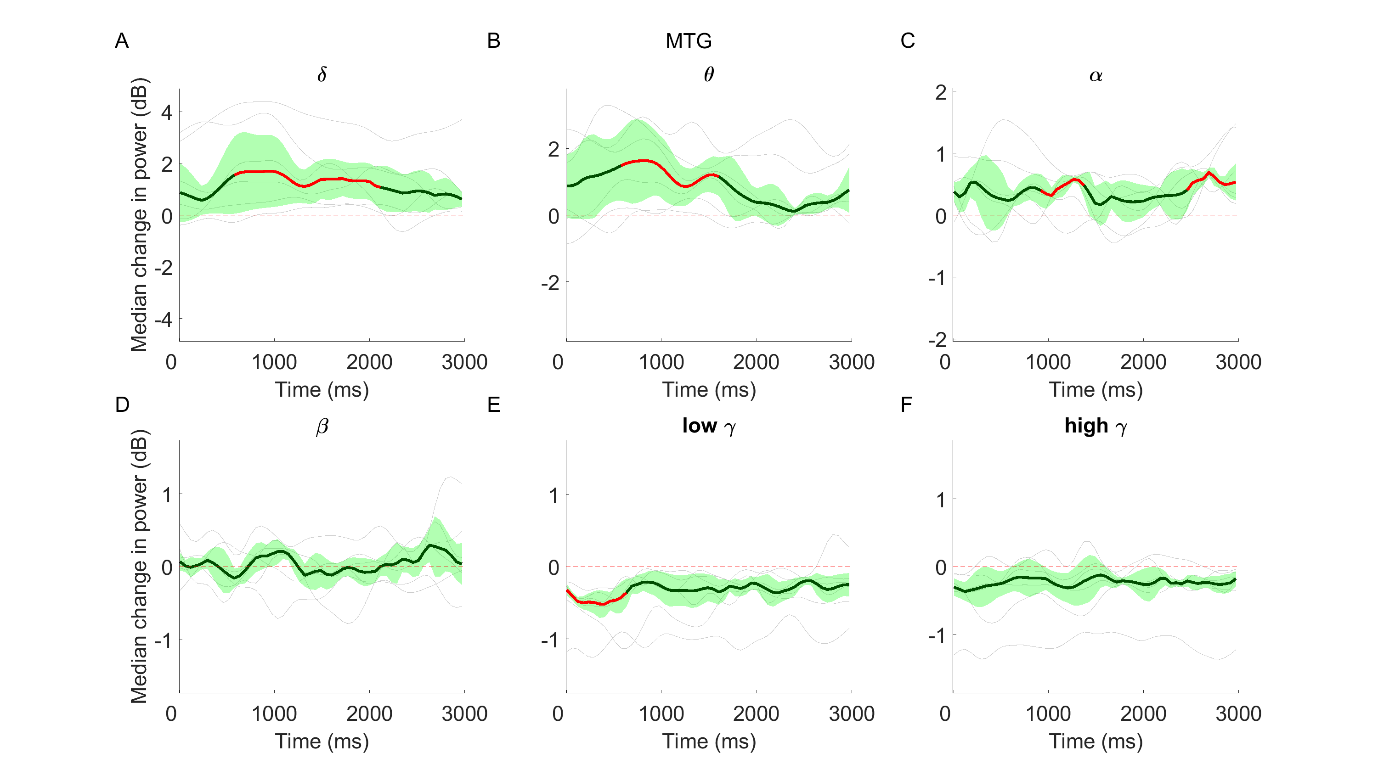
**

**Figure S8.** Median change (± MAD) in power across subjects, relative to baseline, plotted across the entire delay period for MTG. Red areas highlight periods of significance.
